# Combinatorial Effect of Non-Steroidal Anti-inflammatory Drugs and NF-κB Inhibitors in Ovarian Cancer Therapy

**DOI:** 10.1371/journal.pone.0024285

**Published:** 2011-09-12

**Authors:** Luiz F. Zerbini, Rodrigo E. Tamura, Ricardo G. Correa, Akos Czibere, Jason Cordeiro, Manoj Bhasin, Fernando M. Simabuco, Yihong Wang, Xuesong Gu, Linglin Li, Devanand Sarkar, Jin-Rong Zhou, Paul B. Fisher, Towia A. Libermann

**Affiliations:** 1 Medical Biochemistry Division, Faculty of Health Sciences, International Center for Genetic Engineering and Biotechnology (ICGEB), University of Cape Town, Cape Town, South Africa; 2 Sanford-Burnham Institute for Medical Research, La Jolla, California, United States of America; 3 Department of Haematology, Oncology and Clinical Immunology, Heinrich Heine-University, Düsseldorf, Germany; 4 BIDMC Genomics and Proteomics Center, Beth Israel Deaconess Medical Center and Harvard Medical School, Boston, Massachusetts, United States of America; 5 Department of Surgery, Beth Israel Deaconess Medical Center and Harvard Medical School, Boston, Massachusetts, United States of America; 6 Department of Human and Molecular Genetics, School of Medicine, VCU Institute of Molecular Medicine, VCU Massey Cancer Center, Virginia Commonwealth University, Richmond, Virginia, United States of America; Enzo Life Sciences, Inc., United States of America

## Abstract

Several epidemiological studies have correlated the use of non-steroidal anti-inflammatory drugs (NSAID) with reduced risk of ovarian cancer, the most lethal gynecological cancer, diagnosed usually in late stages of the disease. We have previously established that the pro-apoptotic cytokine melanoma differentiation associated gene-7/Interleukin-24 (*mda*-7/IL-24) is a crucial mediator of NSAID-induced apoptosis in prostate, breast, renal and stomach cancer cells. In this report we evaluated various structurally different NSAIDs for their efficacies to induce apoptosis and *mda*-7/IL-24 expression in ovarian cancer cells. While several NSAIDs induced apoptosis, Sulindac Sulfide and Diclofenac most potently induced apoptosis and reduced tumor growth. A combination of these agents results in a synergistic effect. Furthermore, *mda*-7/IL-24 induction by NSAIDs is essential for programmed cell death, since inhibition of *mda*-7/IL-24 by small interfering RNA abrogates apoptosis. *mda*-7/IL-24 activation leads to upregulation of growth arrest and DNA damage inducible (GADD) 45 α and γ and JNK activation. The NF-κB family of transcription factors has been implicated in ovarian cancer development. We previously established NF-κB/IκB signaling as an essential step for cell survival in cancer cells and hypothesized that targeting NF-κB could potentiate NSAID-mediated apoptosis induction in ovarian cancer cells. Indeed, combining NSAID treatment with NF-κB inhibitors led to enhanced apoptosis induction. Our results indicate that inhibition of NF-κB in combination with activation of *mda*-7/IL-24 expression may lead to a new combinatorial therapy for ovarian cancer.

## Introduction

Ovarian cancer represents the most lethal gynecological cancer and the 5^th^ leading cause of women death related to cancer in the United States [1]. Late diagnosis is one of the main hurdles to treat ovarian cancer, as nearly 70% of women present with an advanced stage of the disease at diagnosis [2]. Several epidemiological studies have suggested that the use of NSAIDs at clinically relevant concentrations reduces colorectal [3], breast [4] and ovarian cancer risks [5], [6], [7], although controversy still remains [8], [9]. One major target of NSAID action is inhibition of cyclooxygenase (COX), which are responsible for the conversion of arachidonic acids into prostaglandins and employ a variety of different mechanisms. The two COX genes, COX-1 and COX-2, are almost identical; however, one relevant difference is that COX-1 expression is constitutive, whereas COX-2 expression is induced by growth factors and pro-inflammatory stimuli [10]. NSAIDs are typically classified as specific COX-2 inhibitors or non-specific COX inhibitors. In ovarian cancer COX-1, but not COX-2 has been found to be overexpressed [11], [12], nevertheless other studies reported that COX-2 is also upregulated [13], [14]. High COX-1 expression in ovarian cancer strongly correlates with high levels of Vascular Endothelial Growth Factor (VEGF) [15], [16], [17] and NSAIDs inhibit VEGF production in ovarian cancer cell lines [12], [18] indicating that COX-1 may regulate VEGF expression. Angiogenesis and VEGF expression are implicated in ascites formation [19] and metastasis of ovarian cancer [20], while its inhibition prevents ascites formation and inhibits disseminated cancer growth [21].

Our previous study demonstrates that NSAIDs also induce apoptosis of cancer cells via induction of *mda*-7/IL-24 expression [22], leading to enhanced expression of two members of the Growth Arrest and DNA-Damage 45 (GADD45) family [23]. The GADD45 gene family encodes three structurally highly related growth arrest- and DNA damage-inducible proteins, GADD45 α, β and γ play a role in the G2/M checkpoint in response to DNA damage [24]. Under normal physiological conditions, *mda*-7/IL-24 is expressed in cells of the immune system and normal melanocytes [25]. High levels of *mda*-7/IL-24 have been demonstrated to specifically induce apoptosis of cancer cells and therefore *mda*-7/IL-24 has been referred as a “magic bullet” [26], [27]. Moreover, several studies indicated that overexpression of *mda*-7/IL-24 by a recombinant adenovirus results in cancer cell apoptosis and therapeutic benefits in ovarian cancer [28], [29].

NF-κB/IκB signaling is another pathway that has been implicated in drug resistance and survival in ovarian cancer [30], [31]. The NF-κB/IκB pathway is emerging as key player in tumorigenesis, invasion and metastasis for various cancers [30], [31] and is a critical step for cancer cells to escape programmed cell death [32]. Furthermore, resistance of cancer cells to chemotherapeutic agents has been associated with deregulated NF-κB activation [33]. Moreover, we have shown that inhibition of constitutively active NF-κB by adenoviral expression of IκB induces apoptosis in prostate cancer cells and inhibits tumor formation in SCID mice [34].

In this report, we compared a broad set of NSAIDs for their efficacies to induce apoptosis of ovarian cancer cells. Since our previous study demonstrated a role for *mda*-7/IL-24 in NSAID-mediated cell death, we also evaluated whether apoptosis induction by NSAIDs is due to induction of *mda*-7/IL-24 expression. Viral delivery of *mda*-7/IL-24 is currently used in clinical trials for various cancers [27]. Identification of drugs that are most efficient in *mda*-7/IL-24 induction may provide an alternative to viral delivery for exploiting the anti-neoplastic effects of *mda*-7/IL-24. We also hypothesized that combining NSAIDs with inhibitors of the NF-κB pathway may enhance the effects of NSAIDs against ovarian cancer. We, therefore, tested pharmacological inhibitors of the NF-κB pathway for their abilities to induce apoptosis in ovarian cancer cells. Here, we demonstrate that Sulindac Sulfide and Diclofenac are the most potent NSAIDs that induce ovarian cancer apoptosis via *mda*-7/IL-24 expression and also reduce tumor growth *in vivo*. *mda* -7/IL-24 expression leads to GADD45α and γ upregulation and JNK kinase activation. Several pharmacological NF-κB inhibitors also induce apoptosis of ovarian cancer cells and in combination with NSAIDs potentiate the apoptotic effect of NSAIDs.

## Results

### NSAIDs are potent inducers of *mda*-7/IL-24 and apoptosis in ovarian cancer cells

**Figure 1:**
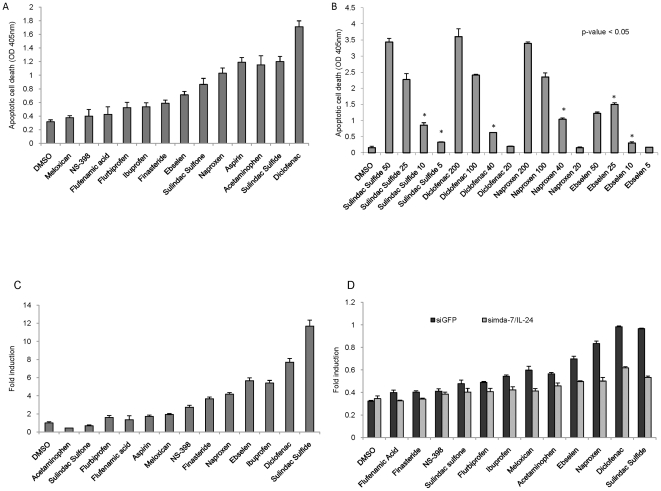
Multiple NSAIDs induce apoptosis and *mda*-7/IL-24 gene expression in ovarian cancer cells. SKOV-3 cell line after treatment with 5 mM Aspirin, 200 µM Ibuprofen, 1 mM Acetaminophen, 200 µM Naproxen, 200 µM NS-398, 200 µM Diclofenac, 50 µM Finasteride, 200 µM Flufenamic acid, 40 µM Meloxicam, 50 µM Ebselen, 20 nM Flurbiprofen, 50 µM Sulindac Sulfide and 50 µM Sulindac Sulfone or DMSO as control. (A) Apoptosis assay of ovarian cancer cells after NSAID treatment. Data means ± s.d. of triplicate independent experiments for each treatment. (B) Dose-dependent induction of apoptosis by NSAIDs in ovarian cancer cells. Apoptosis assay of SW626 ovarian cancer cells. Cells were treated with 50, 25, 10 and 5 µM of Sulindac Sulfide; 200, 100, 40 and 20 µM of Diclofenac; 200, 100, 40 and 20 µM of Naproxen and 50, 25, 10 and 5 µM of Ebselen or DMSO. Apoptosis was measured 24 hours post-treatment. Data means ± s.d. of triplicate independent experiments for each treatment. (C) Real time PCR analysis of *mda*-7/IL-24 expression in SKOV-3 cells after 24 hours treatment with different NSAIDs. Each sample was normalized to hGAPDH. (D) Apoptosis assay of CAOV-3 ovarian cancer cells after NSAID treatment or DMSO and infection with lentivirus encoding *mda*-7/IL-24 siRNA duplexes. Data means ± s.d. of triplicate independent infection for each vector at each treatment.

A broad panel of NSAIDs was tested for their abilities to induce apoptosis and *mda-*7/IL-24 gene expression in four ovarian cancer cell lines, SKOV-3, CAOV-3, SW626 and 36M2. The concentrations for all NSAIDs drugs used in this study were selected to be comparable to achievable physiological plasma concentrations [35]–[49]. Apoptosis was measured 24 hours after treatment of SKOV-3, CAOV-3, SW626 and 36M2 ovarian cancer cells with this set of NSAIDs, revealing that a variety of, but not all NSAIDs induced apoptosis (Figure 1a and S1a). Consistently strong inducers of apoptosis in all four-cell lines included Sulindac Sulfide, Diclofenac, Ebselen and Naproxen when compared to the solvent controls. Some NSAIDs resulted in significant induction of apoptosis in a subset of the ovarian cancer cell lines including Sulindac Sulfone, Acetaminophen, Aspirin and Flurbiprofen whereas treatment with NS-398, Ibuprofen, Finasteride, Flufenamic Acid and Meloxicam resulted only in marginal or no apoptosis induction (Figure 1a and S1a).

For each of the four most consistent inducers of apoptosis we determined the lowest dose that still induces programmed cell death of ovarian cancer cells. The concentrations of the selected NSAIDs were tested at 2, 5 and 10 times lower concentrations than the physiologically achievable doses used in the experiments for Figure 1a and S1a. Apoptosis was measured in ovarian cancer cells 24 hours after treatment with different doses of the four NSAIDs. Our results show that Sulindac Sulfide, Diclofenac and Naproxen concentrations even at 5 times lower dose still effectively induce apoptosis, while Ebselen can be reduced only 2-fold (Figure 1b).

Based on our previous observations in other types of cancer we determined whether apoptosis induction by NSAIDs correlates with *mda*-7/IL-24 induction. We measured mRNA expression levels of *mda-*7/IL-24 mRNA in response to different NSAIDs in SKOV-3 cells by real time PCR analysis demonstrating that *mda-*7/IL-24 (maximum of 12-fold induction) expression is commonly induced by NSAIDs that promote apoptosis in ovarian cancer cells (Figure 1c). These results were also corroborated in two additional ovarian cancer cell lines. In CAOV-3 and SW626 ovarian cancer cell lines *mda-*7/IL-24 is also strongly induced (maximum of 77-fold induction in CAOV-3 cells) (Figure S1b). NSAIDs that strongly enhanced apoptosis such as Sulindac Sulfide, naproxen, ebselen, and diclofenac (Figure 1a) significantly induced *mda*7/IL-24 expression (Figure 1c), whereas NSAIDs that only marginally induced apoptosis (Figure 1a) did not significantly enhance *mda*-7/IL-24 expression except ibuprofen which induced *mda*-7/IL-24 but did not induce apoptosis (Figure 1c).

We and others have shown that overexpression of *mda*-7/IL-24 following infection with an adenovirus carrying the *mda*-7/IL-24 gene induces apoptosis and inhibits cell proliferation in cancer cells [50], [51]. In order to evaluate whether induction of apoptosis in cancer cells by NSAIDs is dependent on *mda*-7/IL-24 upregulation, we conducted experiments with a lentivirus encoding a siRNA against *mda*-7/IL-24 that was previously generated by our group [23]. Infection of SKOV-3 cells with the *mda*-7/IL-24 siRNA lentivirus reduced apoptosis induced by NSAIDs by 40–70% relative to the control lentivirus (Figure 1d), further supporting the notion that NSAID-mediated apoptosis is at least partially dependent on *mda*-7/IL-24 induction. The same results were observed in CAOV-3 cells (data not shown).

### Synergistic effects of NSAID combinations

**Figure 2:**
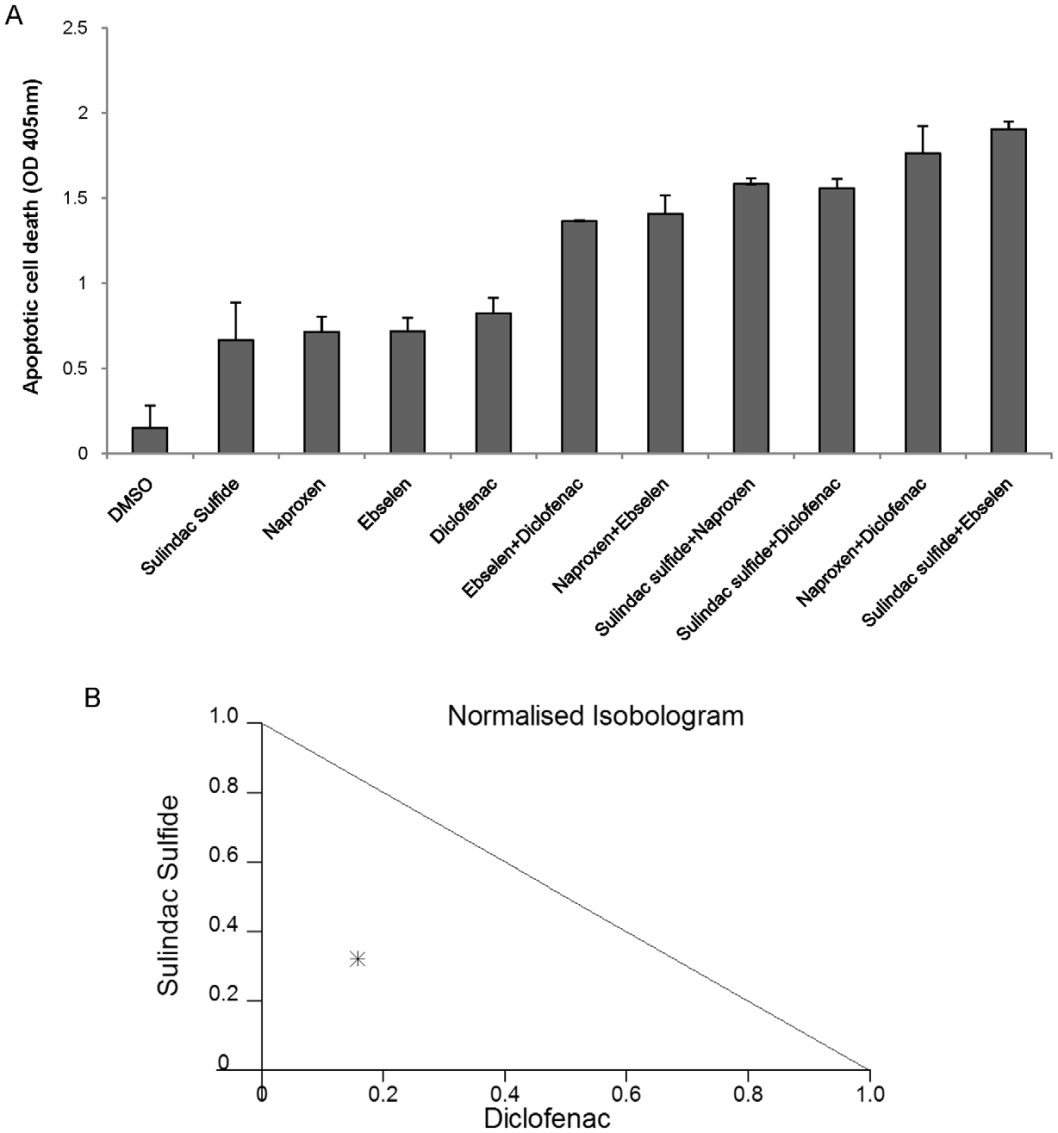
Synergistic effects of NSAID used in combinations. (A) Apoptosis assay of SKOV-3 cells after treatment with 10 µM Sulindac Sulfide, 40 µM Diclofenac, 25 µM Ebselen or 40 µM Naproxen and a combination thereof or DMSO. Apoptosis was measured 24 hours post-treatment. Data means ± s.d. of triplicate independent experiments for each treatment. (B) Normalised isobologram obtained by software Compusyn. CAOV-3 cells treated with a combination of 5 µM Sulindac Sulfide and 20 µM Diclofenac shows synergistic effect.

Using the lowest dose of each NSAID that affected apoptosis of ovarian cancer cells (Figure 1b), we systematically analyzed apoptosis induction upon combining low doses of NSAIDs. A panel of NSAIDs including Diclofenac, Sulindac Sulfide, Naproxen and Ebselen were tested for their abilities to induce apoptosis alone and in combination. SKOV-3 ovarian cancer cells were treated with 10 µM Sulindac Sulfide, 40 µM Diclofenac, 25 µM Ebselen or 40 µM Naproxen and combinations thereof. Apoptosis was measured 24 hours after treatment revealing that the majority of the combinations of NSAIDs tested induced apoptosis significantly more than either of the NSAIDs alone in ovarian cancer cells (Figure 2a). Certain combinations such as Sulindac Sulfide and Diclofenac, Sulindac Sulfide and Naproxen and Diclofenac and Naproxen were more effective in apoptosis induction than others (Figure 2a). We validated these results in CAOV-3 and SW626 cell lines (Figure S2). Isobologram analysis using combinations of Diclofenac and Sulindac Sulfide indicates that combinations of the drugs results in a synergistic effect (Figure 2b).

### NSAID treatment reduces ovarian cancer xenograft growth in SCID mice

**Figure 3:**
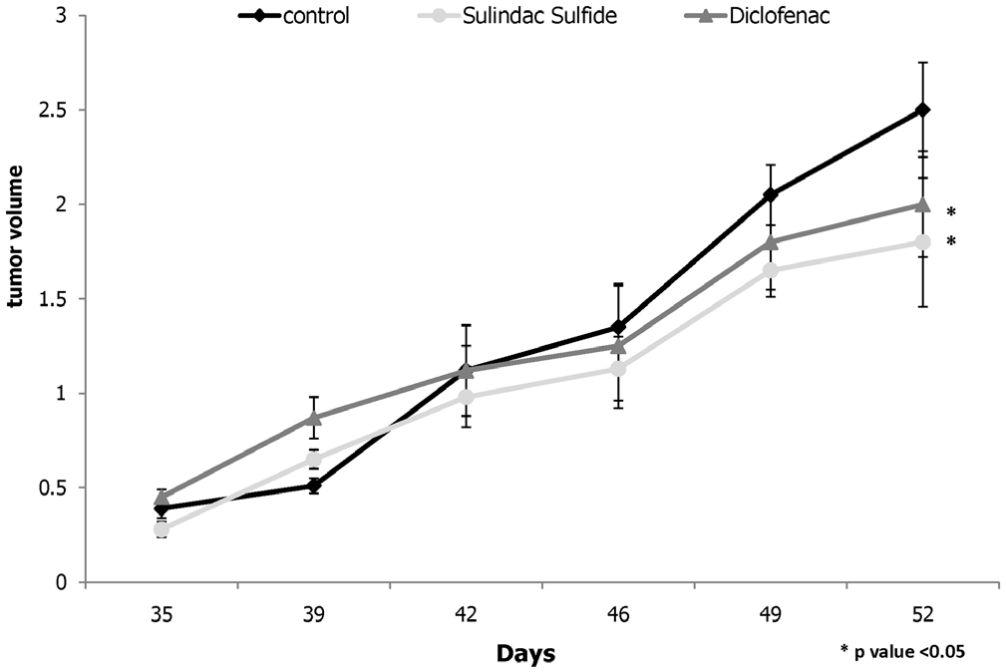
Reduction of tumor formation by NSAIDs. For each inoculation, 2×10^6^ SKOV-3 cells were injected subcutaneously into SCID mice. The mice were randomly divided into three groups (n = 7/group) and fed one of three diets through the entire experiment: AIN-93G as the control, the AIN-93G diet supplemented with 200 ppm Sulindac Sulfide or the AIN-93G diet supplemented with 100 ppm Diclofenac. The size of the tumors and tumor weight were measured after 2 months. Values not sharing the same letters are statistically significant with p values at least <0.05.

To determine whether NSAIDs reduce tumor growth *in vivo*, SKOV-3 ovarian cancer cells were injected subcutaneously in SCID mice. The mice were randomly divided into 3 groups and fed one of three diets through the entire experiment: AIN-93G as the control and the AIN-93G diet supplemented with either 200ppm Sulindac Sulfide or 100ppm Diclofenac. Two months later the animals were examined for tumor formation and tumor weight. All mice developed tumors indicating that this particular dose of NSAIDs did not prevent tumor formation. However, as seen in Figure 3, Sulindac Sulfide and Diclofenac treatment reduced the average tumor volume by 30% and 20%, respectively, when compared to the control diet with a p-value <0.05, confirming its anti-tumor efficacy.

### Induction of GADD45 α and γ gene expression and activation of JNK in ovarian cancer by NSAIDs

We have previously reported that induction of apoptosis by NSAIDs is tightly linked to induction of *mda*-7/IL-24 expression and consequently to GADD45 α and γ upregulation in several cancer cell lines [23]. As NSAIDs induce *mda*-7/IL-24 expression in ovarian cancer cells (Figure 1c and S1c), we evaluated changes in GADD45 α and γ gene expression.

To evaluate whether regulation of GADD45 genes is involved in NSAID-mediated apoptosis, expression of GADD45 α and γ mRNAs was measured by real time PCR in SKOV-3, CAOV-3, and SW626 cells treated with NSAIDs. The NSAIDs with the strongest pro-apoptotic activity including Sulindac Sulfide, Diclofenac, Naproxen and Ebselen strongly enhanced GADD45 α and γ expression, indicating that increased GADD45 α and γ expression closely correlates with pro-apoptotic activity of NSAIDs (Figure S3a and S3b). Lentiviral expression of siRNA against *mda*-7/IL-24 in ovarian cancer cells demonstrated that knockdown of *mda*-7/IL-24 reduces diclofenac-induced GADD45 α and γ gene expression indicating that GADD45α and γ induction is at least partially dependent on *mda*-7/IL-24 expression (Figure S3c).

**Figure 4:**
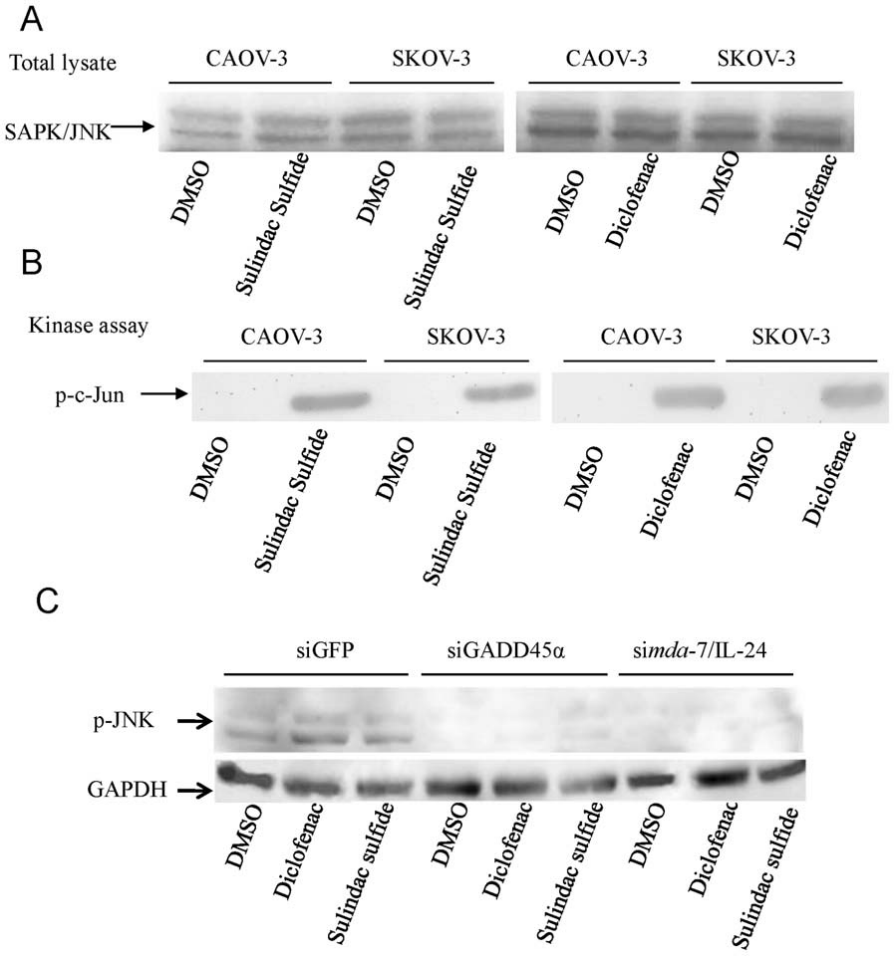
NSAID-treatment induces JNK activation. (A) Total lysate before Immunoprecipitation. (B) Kinase assay showing induction of JNK kinase activity by NSAIDs. Induction of JNK activation by Sulindac Sulfide and Diclofenac was analyzed in cell lysates from SKOV-3 and CAOV-3 cells treated with 50 µM Sulindac Sulfide, 100 µM Diclofenac or DMSO using the SAPK/JNK assay Kit (Cell Signaling). (C) Western Blot analysis using anti-phospho JNK antibody of cell lysates from CAOV-3 cells treated with 50 µM Sulindac Sulfide, 100 µM Diclofenac or DMSO and infection with lentivirus encoding *mda*-7/IL-24 siRNA, GADD45α and GFP duplexes.

Since others and we had shown that JNK activation plays a role in apoptosis induction in cancer cells and GADD45 α and γ interact with the upstream kinase of JNK, MTK1 [52], we evaluated the activation of JNK during NSAID-mediated apoptosis. JNK kinase activity was tested in protein extracts obtained from CAOV-3 and SKOV-3 cells treated with Sulindac Sulfide (50 µM), Diclofenac (200 µM) or DMSO for 24 hours by an *in vitro* kinase assay. Western blot analysis revealed very little JNK activity in untreated control cells and a strong increase in JNK activity in both cell lines upon treatment with Sulindac Sulfide and Diclofenac (Figure 4a and 4b). Corroborating this evidence a weak inducer of *mda*-7/IL-24, GADD45 α and γ and apoptosis, Flurbiprofen, demonstrated only a marginal induction of JNK activity (data not shown). To elucidate the functional relevance of GADD45 α and γ and *mda*-7/IL-24 for NSAID-mediated JNK induction and apoptosis in ovarian cancer, JNK kinase activity was tested in protein extracts obtained from *CAOV-3* cells treated with Sulindac Sulfide and Diclofenac and infected with lentivirus encoding siRNA against GADD45 α and *mda*-7/IL-24 genes. Western blot analysis revealed JNK kinase activation by Sulindac Sulfide and Diclofenac was markedly dependent on GADD45 α and *mda*-7/IL-24 induction, since JNK kinase activity in Sulindac Sulfide and Diclofenac treated *mda*-7/IL-24-/- cells was abolished when compared to *mda*-7/IL-24+/+ cells (Figure 4b). In order to further characterize the effect of NSAIDS in inducing apoptosis, the levels of PARP activation were measured by Western-blot, indicating that Sulindac Sulfide and Diclofenac are strong inducers of PARP cleavage (Figure S4a).

### Combinatorial treatment of pharmacological inhibitors of the NF-κB pathway with NSAIDs induce apoptosis in ovarian cancer cells

**Figure 5:**
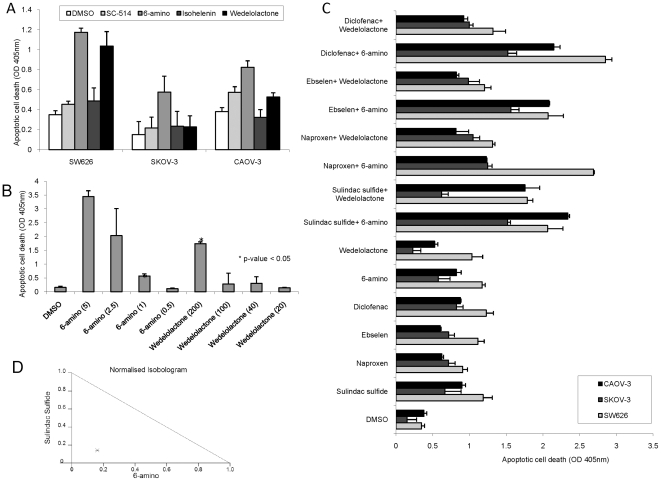
Combinatorial treatment of ovarian cancer cells with NSAIDs and NF-κB inhibitors. (A) Pharmacological NF-κB inhibitors induce apoptosis in ovarian cancer cells. SW626, CAOV-3, and SKOV-3 cells after treatment with 5 nM 6-Amino-4-(4-phenoxyphenylethylamino) quinazoline (6-amino), 50 µM Isohelenin, 50 µM IKK-2 inhibitor SC-514, and 200 µM IKK inhibitor II Wedelolactone (7-Methoxy-5,11,12-trihydroxy-coumestan) or DMSO as control. Data means ± s.d. of triplicate independent experiments for each treatment. (B) Dose-dependent induction of apoptosis by NF-κB inhibitors in ovarian cancer cells. Apoptosis assay of SKOV-3 ovarian cancer cells. Cells were treated with 5, 2.5, 1 and 0.5 nM of 6-Amino-4-(4-phenoxyphenylethylamino) quinazoline (6-amino) 50, 25, 10 and 5 µM of Isohelenin, 50, 25, 10 and 5 µM of IKK inhibitor II Wedelolactone (7-Methoxy-5,11,12-trihydroxy-coumestan) (Wedelolactone), 50, 25, 10 and 5 µM of IKK-2 inhibitor SC-514 or DMSO. Apoptosis was measured 24 hours post-treatment. Data means ± s.d. of triplicate independent experiments for each treatment. (C) Apoptosis in ovarian cancer cells after NSAID treatment in combination with NF-κB inhibitors. SW626, CAOV-3, and SKOV-3 cells after treatment with 10 µM Sulindac Sulfide, 40 µM Diclofenac, 25 µM Ebselen, 40 µM Naproxen, 1 nM 6-Amino-4-(4 phenoxyphenylethylamino) quinazoline (6-amino), and 200 µM IKK inhibitor II Wedelolactone and a combination thereof. Apoptosis was measured 24 hours after treatment. Data means ± s.d. of triplicate independent experiments for each treatment. (D) Normalised isobologram obtained by software Compusyn. CAOV-3 cells treated with a combination of 10 µM Sulindac Sulfide and 2.5 nM 6-amino shows synergistic effect.

We investigated the biological relevance of the NF-κB pathway in ovarian cancer cells and determined the functional consequences of its inhibition. Instead of using adenoviral delivery of the IκB inhibitor we moved towards a more clinically relevant model and used pharmacological inhibitors of the NF-κB pathway. Inhibitors of the NF-κB pathway were tested for their abilities to induce apoptosis in ovarian cancer cells. Apoptosis was measured 24 hours after treatment of SKOV-3, CAOV-3 and SW626 ovarian cancer cells with four different inhibitors of NF-κB, 5 nM 6-Amino-4-(4-phenoxyphenylethylamino) quinazoline [53], 50 µM Isohelenin [54], 50 µM IKK-2 inhibitor SC-514 [55], and 200 µM IKK inhibitor II Wedelolactone (7-Methoxy-5,11,12-trihydroxy-coumestan) [56] or DMSO (control). 6-Amino-4-(4-phenoxyphenylethylamino) quinazoline was an efficient inducer of apoptosis in all three cell lines, and the IKK inhibitor II Wedelolactone (7-Methoxy-5,11,12-trihydroxy-coumestan) induced apoptosis in two of the three cell lines. Treatment with Isohelenin or IKK-2 inhibitor SC-514 resulted only in marginal or no apoptosis induction (Figure 5a). Additionally, Real-time PCR analysis indicates that Wedelolactone (7-Methoxy-5,11,12-trihydroxy-coumestan) induces strong activation of the GADD45 α and γ gene expression (Figure S4b), and promotes JNK phosphorylation (Figure S4c) and cleavage of PARP (Figure S4a).

As for the NSAIDs we performed a dose response analysis for 6-Amino-4-(4-phenoxyphenylethylamino) quinazoline and Wedelolactone (7-Methoxy-5,11,12-trihydroxy-coumestan) to determine the lowest dose that still induces programmed cell death of ovarian cancer cells. Reducing the concentration of 6-Amino-4-(4-phenoxyphenylethylamino) quinazoline from 5 nM to 1 nM still induced apoptosis, while reduced doses of Wedelolactone resulted in loss of apoptosis induction (Figure 5b).

To determine whether the NF-κB inhibitors 6-Amino-4-(4-phenoxyphenylethylamino) quinazoline and Wedelolactone (7-Methoxy-5,11,12-trihydroxy-coumestan) enhance the pro-apoptotic activities of NSAIDs we combined the lowest doses of each of the four NSAIDs, Sulindac Sulfide, Diclofenac, Ebselen, and Naproxen with the lowest doses of the two NF-κB inhibitors that still induce apoptosis (Figure 1b and 5b, respectively). The NSAIDs and NF-κB inhibitors were tested for their abilities to induce apoptosis alone and in combination. SKOV-3, CAOV-3, and SW626 ovarian cancer cells were treated with 10 µM Sulindac Sulfide, 40 µM Diclofenac, 25 µM Ebselen, 40 µM Naproxen, 1 nM 6-Amino-4-(4 phenoxyphenylethylamino) quinazoline and 200 µM Wedelolactone. Apoptosis was measured 24 hours after treatment revealing that combinations of NSAIDs with the NF-κB inhibitor 6-Amino-4-(4 phenoxyphenylethylamino) quinazoline significantly enhanced apoptosis in ovarian cancer cells compared to either of the drugs alone (Figure 5c). In contrast, Wedelolactone enhanced the pro-apoptotic effects only of some of the NSAIDs and less efficiently in SKOV-3 cells (Figure 5c). Isobologram analysis indicates that the combination of 6-Amino-4-(4 phenoxyphenylethylamino) quinazoline with Sulindac Sulfide results in a synergistic effect (Figure 5d).

## Discussion

NSAIDs have emerged as potential drugs for chemoprevention in cancer but their benefits are still in question. Some traditional NSAIDS such as Sulindac are currently being tested in clinical trials for various cancers. Indeed, preclinical studies provide consistent evidence that NSAIDs can effectively inhibit tumorigenesis in particular through inhibition of cyclooxygenase-2 (COX-2). Importantly, aspirin use has been associated with a decreased risk of distant breast cancer recurrence and breast cancer death [57].

Epidemiologic studies indicate inverse associations between use of nonsteroidal anti-inflammatory drugs (NSAID) and the incidence of ovarian cancer [5], [6], [7]. Several reports suggest that certain NSAIDs induce apoptosis and cell cycle arrest in human ovarian cancer cells but the exact molecular mechanism by which NSAIDs induce antitumorigenic activity is not clear. Previously, our groups described a novel pathway by which NSAIDs induce apoptosis and growth arrest in cancer cells. We demonstrated that induction of the pro-apoptotic cytokine MDA-7/IL-24 by NSAIDs is crucial for programmed cell death induced by NSAIDs [23]. However, this study did not include ovarian cancer cells.

Several reports using an adenovirus encoding the *mda*-7/IL-24 gene (Ad-*mda*-7) show its profound and selective anticancer activity in animal models [26], [27], [50], [58], [59], [60] including a report of selectively induced cell death of ovarian cancer cells that results in suppression of tumor growth *in vivo* [58]. However, transient expression, potentially adverse immune reactions (mediated by adenovirus) and problems with systemic delivery restrict the generalized use of adenoviral delivery of *mda*-7/IL-24, particularly when administered systemically as a non-replicating adenovirus.

In this context, our findings that NSAIDs with anti-cancer activity induce high levels of *mda*-7/IL-24 in ovarian cancer cells provide a new therapeutic strategy to enhance *mda*-7/IL-24 levels on a systemic level. Indeed, we have obtained a comprehensive overview of the consequences of a whole panel of NSAIDs on ovarian cancer cell survival by comparing their efficacies to induce apoptosis and *mda*-7/IL-24 expression. The most potent inducers of *mda*-7/IL-24 gene expression include Sulindac Sulfide and Diclofenac. Our finding corresponds with previous reports that demonstrated that treatment of human lung tumor xenografts in nude mice with Ad-*mda*-7 in addition to Sulindac reduced tumor growth more efficiently than Ad-*mda*-7 [50]. Moreover, these results corroborate our previous findings that apoptosis induction of the pro-apoptotic cytokine *mda*-7/IL-24 mediates induction of GADD45 α and γ expression and JNK activity in other types of cancer [23]. While Sulindac Sulfide and Diclofenac themselves may not be the ideal drugs to induce *mda*-7/IL-24 and apoptosis in ovarian cancer cells, and particularly Diclofenac elicits many adverse effects in patients that limit its use in cancer patients, it should be feasible to generate modified versions of these drugs that are more potent in their anti-cancer activities and with reduced adverse and off-target effects. Indeed a modified version of Sulindac has recently been reported to be more active against cancer cells without inhibiting COX 1 and 2 [61].

Diclofenac has previously been shown to induce apoptosis in colon and squamous cell carcinoma and to inhibit pancreatic tumor growth [62], [63]. However, there are no reports about its use in ovarian cancer. Here, we demonstrate that Diclofenac as well as Sulindac Sulfide induce apoptosis and inhibit tumor growth of ovarian cancer. These compelling data reinforce the notion of the potential benefits of NSAID treatment for ovarian cancer.

We also identified Naproxen and Ebselen as moderate inducers of apoptosis and *mda*-7/IL-24 expression in ovarian cancer cells. While Naproxen helps to prevent urinary bladder and colon carcinogenesis [64], Ebselen has been shown to reduce cisplatin treatment toxicity in rat ovarian cancer models, enhancing anti-tumor activity and improving mortality, morbidity and outcome [65]. As mentioned before, we have reported that induction of *mda*-7/IL-24 by structurally different NSAIDs is crucial for apoptosis induction of breast, prostate, renal and stomach cancer cells [23]. However, in this previous study, Naproxen and Ebselen had only marginal effects on apoptosis induction. In this report, we observed different drug activities for Naproxen and Ebselen. Ebselen and Naproxen induced apoptosis and *mda*-7/IL-24 expression in ovarian cancer cells and also synergized with the more potent NSAIDs, Diclofenac and Sulindac Sulfide, suggesting potential clinical utility in ovarian cancer therapy.

We have previously shown that inhibition of NF-κB in cancer cells increases apoptosis without promoting *mda*-7/IL-24 production [23]. One of the major transcriptional circuits implicated in inflammation is the NF-κB/IκB pathway [66]. Furthermore, NF-κB has been implicated in cancer cell survival and escape from programmed cell death and is activated by chemotherapeutic agents in cancer cells [30], [31], [33]. Mutations in different genes of the NF-κB pathway and constitutively active NF-κB are frequently observed in various types of cancer [33]. Indeed, ovarian cancer cells frequently contain activated NF-κB prior to therapy and are, therefore, expected to be resistant to chemotherapy *a priori*. On the other hand, we have demonstrated that inhibition of activated NF-κB in cancer cells induces apoptosis without the addition of a chemotherapeutic agent indicating the central role of NF-κB in cell survival of many cancer cells. These findings also suggest the possibility of enhancing therapeutic outcomes by combining NF-κB inhibitors with chemotherapy or other drugs such as NSAIDs. At high concentrations, NSAIDs have been shown to inhibit the TNF-mediated activation of NF-κB [67]. However, we have previously shown that at achievable plasma concentrations, NSAIDs Sulindac Sulfide has no effect on the NF-κB signaling pathway [23]. Indeed here we demonstrate that NF-κB inhibitors strongly induce apoptosis in ovarian cancer cells. Importantly, NF-κB inhibitors markedly enhanced the efficacy of NSAIDs to induce apoptosis, corroborating our hypothesis that these combinatorial regimens can be utilized even though animal model studies are still necessary to prove its efficacy *in vivo*.

In summary, our results strongly support the hypothesis that drug treatment regimens that lead to enhanced *mda*-7/IL-24 expression in cancer cells and block NF-κB may have significant efficacy against ovarian cancer. These results also provide a rationale to screen for additional small molecules, natural compounds or even chemically modified NSAIDs, which selectively and efficiently induce *mda*-7/IL-24 expression in order to obtain more potent anti-cancer drugs.

## Materials and Methods

### Cell cultures

The ovarian cancer cell lines SKOV-3, CAOV-3, and SW626 were obtained from American Type Culture Collection (Rockville, MD). 36M2 cells were kindly provided by Dr. Dimitrios Spentzos, Beth Israel Deaconess Medical Center. SKOV-3 were grown in McCoy' 5a modified medium (Life Technologies, Carlsbad, CA), SW626 were grown in L-15 medium; and CAOV-3 and 36M2 were grown in DMEM (Life Technologies, Carlsbad, CA). The medium was supplemented with 10% fetal bovine serum (FBS), 50 units of penicillin/mL, and 50 µg streptomycin/mL (all from Life Technologies). The cells were maintained in a 5% CO_2_-humidified incubator at 37°C.

### Reagents

Sulindac Sulfide, Sulindac Sulfone, Ibuprofen, Aspirin, Acetaminophen, and Naproxen were obtained from Sigma-Aldrich (St. Louis, MO). Meloxicam, Diclofenac, Finasteride, and Flufenamic acid were obtained from LKT Laboratories (St. Paul, MN). NS-398, Ebselen, and Flurbiprofen were purchased from Calbiochem (San Diego, CA). The drugs were dissolved in DMSO or ethanol. Cancer cells were treated in their particular medium for 24 hours. The concentrations of the different NSAIDs were chosen to be physiologically achievable doses. The final concentrations for each compound were as follows: 5 mM Aspirin, 200 µM Ibuprofen, 1 mM Acetaminophen, 200 µM Naproxen, 200 µM NS-398, 200 µM Diclofenac, 50 µM Finasteride, 200 µM Flufenamic acid, 40 µM Meloxicam, 50 µM Ebselen, 20 nM Flurbiprofen, 50 µM Sulindac Sulfide and 50 µM Sulindac Sulfone. For the controls, cells were treated with an equal amount of DMSO or ethanol, which was <0.1% of the final concentration.

NF-κB inhibitors: 5 nM 6-Amino-4-(4-phenoxyphenylethylamino) quinazoline, 50 µM Isohelenin, 50 µM IKK-2 inhibitor SC-514, and 200 µM IKK inhibitor II Wedelolactone (7-Methoxy-5,11,12-trihydroxy-coumestan) were purchased from Calbiochem (San Diego, CA).

### Real-time PCR

Total RNA was harvested using QIAshredder (Qiagen, Valencia, CA) and RNeasy Mini kit (Qiagen). Real-time PCR was performed as described [23]. The full description of the method is described in Methods S1. The sequences of the primers are as follows: *mda*-7/IL-24, sense 5′-CAAAGCCTGTGGACTTTAGCC-3′; antisense 5′-GAATAGCAGAAACCGCCTGTG- 3′; hGAPDH, sense 5′-CAAAGTTGTCATGGATGACC-3′; antisense 5′-CCATGGAGAAGGCTGGGG-3′. GADD45α, sense 5′-GCCTGTGAGTGAGTGCAGAA- 3′; antisense 5′-ATCTCTGTCGTCGTCCTCGT-3′; GADD45γ, sense 5′-CTGCATGAGTTGCTGCTGTC- 3′; antisense 5′-TTCGAAATGAGGATGCAGTG –3′.

### Kinase assay

JNK kinase activity was measured by using the SAPK/JNK assay kit (Cell Signaling Technology) according to the manufacturer' protocol. Briefly, CAOV-3 and 36M2 cells were treated with Sulindac Sulfide, Diclofenac or DMSO for 16 hours. Whole-cell lysates of treated and control cells were prepared in lysis buffer [20 mmol/L Tris (pH 7.4), 150 mmol/L NaCl, 1 mmol/L EDTA, 1 mmol/L EGTA, 1% Triton X-100, 2.5 mmol/L sodium PPi, 1 mmol/L βGlycerophosphate, 1 mmol/L Na_3_VO_4_, 1 µ g/mL leupeptin, and 1 mmol/L phenylmethylsulfonyl fluoride]. 500 µg of the clarified lysate were immunoprecipitated using 2 µg agarose-conjugated anti-c-jun monoclonal antibody (Cell Signaling, Beverly MA) overnight at 4°C. The beads were washed twice with lysis buffer and twice with kinase buffer (25 mM Tris (pH 7.5), 5 mM β-Glycerophosphate, 2 mM DTT, 0.11 mM Na_3_VO_4_, 10 mM MgCl_2_) and subjected to the kinase assays. The beads were suspended in 50 µl kinase buffer supplemented with 200 µM ATP and incubated for 30 minutes at 30°C. The reactions were terminated by adding 25 µl 3X SDS sample buffer and proteins were resolved by SDS-10% PAGE and probed with phospho-c-jun antibody (Cell Signalling).

### Western-blot

Western-blot were performed as described in supplementary information by using antibodies against total SAPK/JNK (New England Biolabs), phospho JNK (Cell Signaling), *mda-7/*IL-24 (kindly provided by Paul B. Fisher, Virginia Commonwealth University, School of Medicine, Richmond, VA), PARP and GAPDH (Santa Cruz Biotechnology).

### Apoptotic assays

Apoptosis was assayed by using the Apoptotic Cell Death Detection ELISA (Roche) according to the manufacturer' protocol. Significant statistical difference of control samples against samples treated with NSAIDs was determined by Student' t-test.

### Isobologram

The isobologram was calculated using Calcusyn software (Biosoft). The drugs were applied across a range of concentrations and cell proliferation was evaluated using the MTT assay at each dosage. Data from cell proliferation was expressed as the fraction of cells inhibited by drug treatments compared with untreated cells. Interaction between pairs of drugs was determined using the Calcusyn software, which generated the isolobogram. The isobologram is a graphical representation of the interaction between two drugs and is formed by plotting the individual drug doses required to achieve a single agent effect on their respective x and y axes, a line connecting the two points is drawn and the concentrations of the two drugs used in combination to achieve the same effect are plotted on the isobologram. Combination data points that fall on the line represent an additive interaction, whereas points above or below represent antagonism or synergy, respectively.

### Lentivirus constructs

The lentiviruses encoding siRNA against the three GADD45 family members have been previously described [34]. The LVsiRNA GFP construct (control) was kindly donated by Dr. Oded Singer (Salk Institute for Biological Studies, San Diego, CA). The lentivirus encoding siRNA against *mda-7*/IL-24 gene was cloned using Advantage 2 PCR kit (Clontech, Mountain View, CA), and the virus was generated by using a previously described methodology [34]. The following siRNA oligonucleotide for *mda-7*/IL-24 was used: 5′CTGTCTAGACAAAAACTTTGTTCTCATCGTGTCATCTCTTGAATGACACGATGAGAACAAAGGGGGATCTGTGGTCTCATACA-3′.

### Animal experiment

Severe combined immunodeficient (SCID)-beige mice were purchased from Taconic (Germantown, NY) and housed in a pathogen-free environment. The animals were randomly divided into 3 groups (n = 7 per group). 8-week-old female SCID-beige mice were fed with one of the experimental diets supplemented with 200 ppm of Sulindac Sulfide, 100 ppm of Diclofenac or control solvents for 2 weeks. Immediately before implantation, SKOV-3 cells were trypsinized and resuspended in MEM with 10% fetal bovine serum. Cell viability was determined by trypan blue exclusion and a single cell suspension with >90% viability was used for implantation. SKOV-3 cells (2×10^6^ cells in 50 µl) were carefully injected subcutaneously as described previously [23], [34] and animals continued on experimental diets. Tumor size (volume) was measured every 5 days, starting at day 35 after implantation. The experiment was finished when the average tumor weight in the control animals reached 2–5% of the body weight. The diets were prepared by Research Diets, Inc. (New Brunswick, NJ). Body weight and food intake were measured weekly. All procedures with animals were reviewed and approved by the Institutional Animal Care and Use Committee at the Beth Israel Deaconess Medical Center according to NIH guidelines.

## Supporting Information

Figure S1 **Multiple NSAIDs induce apoptosis and** ***mda-7*** **/IL-24 gene expression in ovarian cancer cells.** CAOV-3 and SW626 ovarian cancer cell lines after treatment with 5 mM Aspirin, 200 µM Ibuprofen, 1 mM Acetaminophen, 200 µM Naproxen, 200 µM NS-398, 200 µM Diclofenac, 50 µM Finasteride, 200 µM Flufenamic acid, 40 µM Meloxicam, 50 µM Ebselen, 20 nM Flurbiprofen, 50 µM Sulindac Sulfide and 50 µM Sulindac Sulfone or DMSO as control. (A) Apoptosis assay of ovarian cancer cells after NSAID treatment. Data means ± s.d. of triplicate independent experiments for each treatment. (B) Real time PCR analysis of *mda*-7/IL-24 expression in SKOV-3 cells after 24 hours treatment with different NSAIDs. Each sample was normalized to hGAPDH. (C) NSAIDs mediated induction of *mda*-7/IL-24. Western-blot analysis using antibody against *mda*-7/IL-24 (kindly provided by Dr. Paul B. Fisher, Virginia Commonwealth University, School of Medicine) shows that treatment with 50 µM Sulindac Sulfide induces *mda*-7/IL-24 expression in a cell line with silenced GFP control gene, while cells with silenced *mda*-7/IL-24 and treated with 50 µM Sulindac Sulfide shows abrogation of its induced expression mediated by NSAIDs.
(TIF)

Figure S2 **Synergistic effects of NSAID used in combinations.** (A) Apoptosis assay of CAOV-3 and SW626 cells after treatment with 10 µM Sulindac Sulfide, 40 µM Diclofenac, 25 µM Ebselen or 40 µM Naproxen and a combination thereof or DMSO. Apoptosis was measured 24 hours post-treatment. Data means ± s.d. of triplicate independent experiments for each treatment.
(TIF)

Figure S3 **NSAIDS treatment of ovarian cancer cells induces GADD45 family gene expression.** Real time PCR analysis of GADD45α (A) and GADD45γ (B) expression after NSAIDS treatment with 5 mM Aspirin, 200 µM Ibuprofen, 1 mM Acetaminophen, 200 µM Naproxen, 200 µM NS-398, 200 µM Diclofenac, 50 µM Finasteride, 200 µM Flufenamic acid, 40 µM Meloxican, 50 µM Ebselen, 20 nM Flurbiprofen, 50 µM Sulindac Sulfide and 50 µM Sulindac Sulfone or DMSO as control. Total RNA was collected from SKOV-3, CAOV-3 and SW626 cells 24 hours after treatment. Normalization of each sample was carried out by measuring the amount of hGAPDH cDNA. (C) Real time PCR analysis of GADD45 α and γ expression after treatment with 200 µM Diclofenac and infection with lentivirus encoding siRNA against *mda-*7/IL-24 or GFP genes. Total RNA was collected from SKOV-3 cell lines after 24 hours after treatment.
(TIF)

Figure S4 **NSAIDs and NF-κB inhibitor activities in ovarian cancer cells.** (A) NSAIDs and NF-κB inhibitors induce PARP activation. Western Blot analysis using anti PARP antibody (Santa Cruz) of cell lysates from CAOV-3 cells treated with 50 µM Sulindac Sulfide, 100 µM Diclofenac, 200 µM IKK inhibitor II Wedelolactone (7-Methoxy-5,11,12-trihydroxy-coumestan) or DMSO. (B) Real-time PCR analysis of CAOV-3 cells treated with 200 µM IKK inhibitor II Wedelolactone (7-Methoxy-5,11,12-trihydroxy-coumestan) or DMSO shows induced expression of GADD45 α and γ genes, (C) while western Blot analysis using anti-phospho JNK antibody of cell lysates from CAOV-3 cells treated with 200 µM IKK inhibitor II Wedelolactone (7-Methoxy-5,11,12-trihydroxy-coumestan) or DMSO shows activation of JNK.
(TIF)

Methods S1 **Description of Real Time methodology.**
(DOCX)
