# Supplementary figures and images for: Combinatorial Effect of Non-Steroidal Anti-inflammatory Drugs and NF-κB Inhibitors in Ovarian Cancer Therapy
Source: PLoS One. 2011 Sep 12;6(9):e24285. doi: 10.1371/journal.pone.0024285 (PMC3171406; doi:10.1371/journal.pone.0024285)

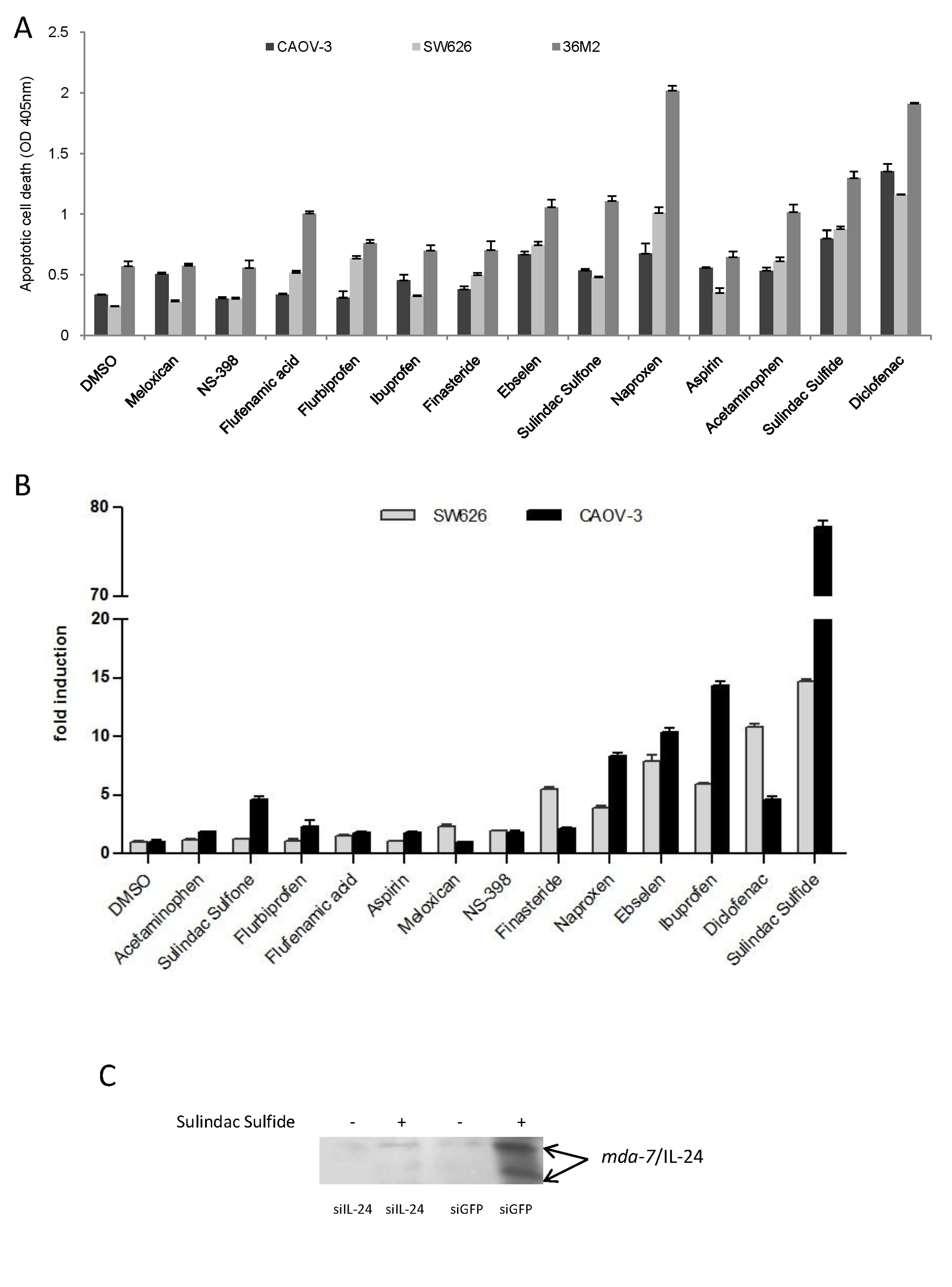

Supplement: Figure S1 — Multiple NSAIDs induce apoptosis and mda-7 /IL-24 gene expression in ovarian cancer cells. CAOV-3 and SW626 ovarian cancer cell lines after treatment with 5 mM Aspirin, 200 µM Ibuprofen, 1 mM Acetaminophen, 200 µM Naproxen, 200 µM NS-398, 200 µM Diclofenac, 50 µM Finasteride, 200 µM Flufenamic acid, 40 µM Meloxicam, 50 µM Ebselen, 20 nM Flurbiprofen, 50 µM Sulindac Sulfide and 50 µM Sulindac Sulfone or DMSO as control. (A) Apoptosis assay of ovarian cancer cells after NSAID treatment. Data means ± s.d. of triplicate independent experiments for each treatment. (B) Real time PCR analysis of mda-7/IL-24 expression in SKOV-3 cells after 24 hours treatment with different NSAIDs. Each sample was normalized to hGAPDH. (C) NSAIDs mediated induction of mda-7/IL-24. Western-blot analysis using antibody against mda-7/IL-24 (kindly provided by Dr. Paul B. Fisher, Virginia Commonwealth University, School of Medicine) shows that treatment with 50 µM Sulindac Sulfide induces mda-7/IL-24 expression in a cell line with silenced GFP control gene, while cells with silenced mda-7/IL-24 and treated with 50 µM Sulindac Sulfide shows abrogation of its induced expression mediated by NSAIDs. (TIF) [file pone.0024285.s001.tif]

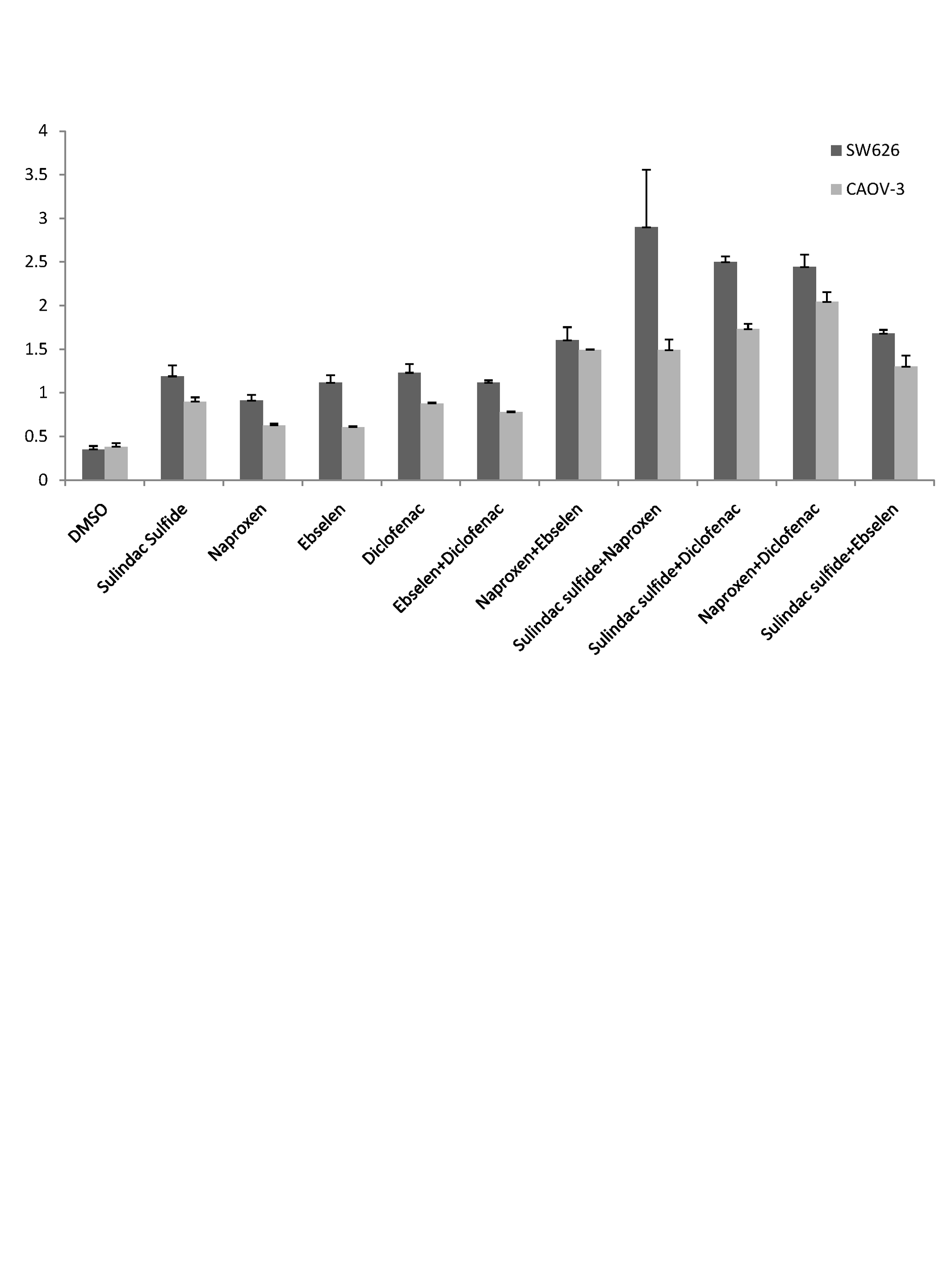

Supplement: Figure S2 — Synergistic effects of NSAID used in combinations. (A) Apoptosis assay of CAOV-3 and SW626 cells after treatment with 10 µM Sulindac Sulfide, 40 µM Diclofenac, 25 µM Ebselen or 40 µM Naproxen and a combination thereof or DMSO. Apoptosis was measured 24 hours post-treatment. Data means ± s.d. of triplicate independent experiments for each treatment. (TIF) [file pone.0024285.s002.tif]

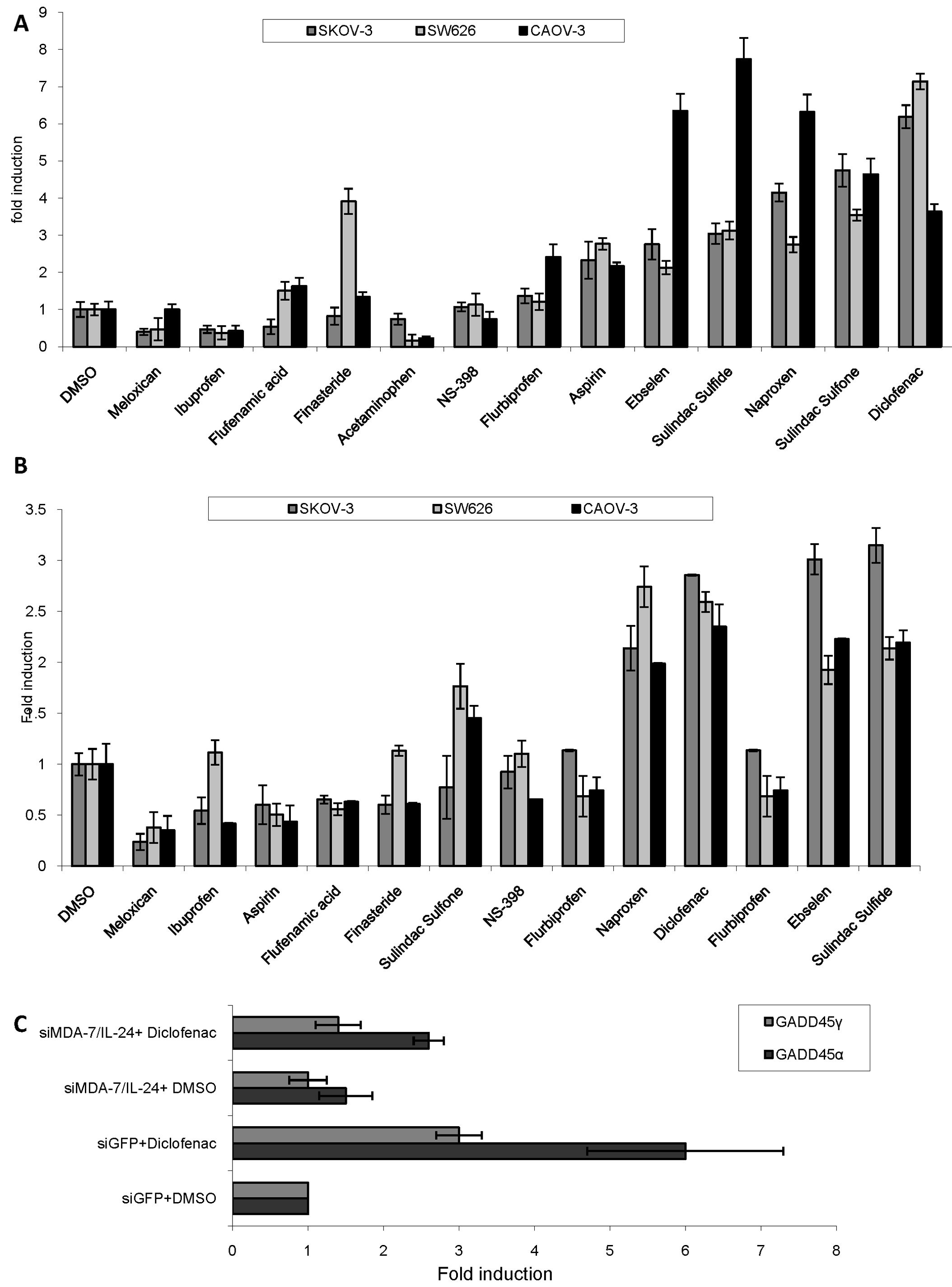

Supplement: Figure S3 — NSAIDS treatment of ovarian cancer cells induces GADD45 family gene expression. Real time PCR analysis of GADD45α (A) and GADD45γ (B) expression after NSAIDS treatment with 5 mM Aspirin, 200 µM Ibuprofen, 1 mM Acetaminophen, 200 µM Naproxen, 200 µM NS-398, 200 µM Diclofenac, 50 µM Finasteride, 200 µM Flufenamic acid, 40 µM Meloxican, 50 µM Ebselen, 20 nM Flurbiprofen, 50 µM Sulindac Sulfide and 50 µM Sulindac Sulfone or DMSO as control. Total RNA was collected from SKOV-3, CAOV-3 and SW626 cells 24 hours after treatment. Normalization of each sample was carried out by measuring the amount of hGAPDH cDNA. (C) Real time PCR analysis of GADD45 α and γ expression after treatment with 200 µM Diclofenac and infection with lentivirus encoding siRNA against mda-7/IL-24 or GFP genes. Total RNA was collected from SKOV-3 cell lines after 24 hours after treatment. (TIF) [file pone.0024285.s003.tif]

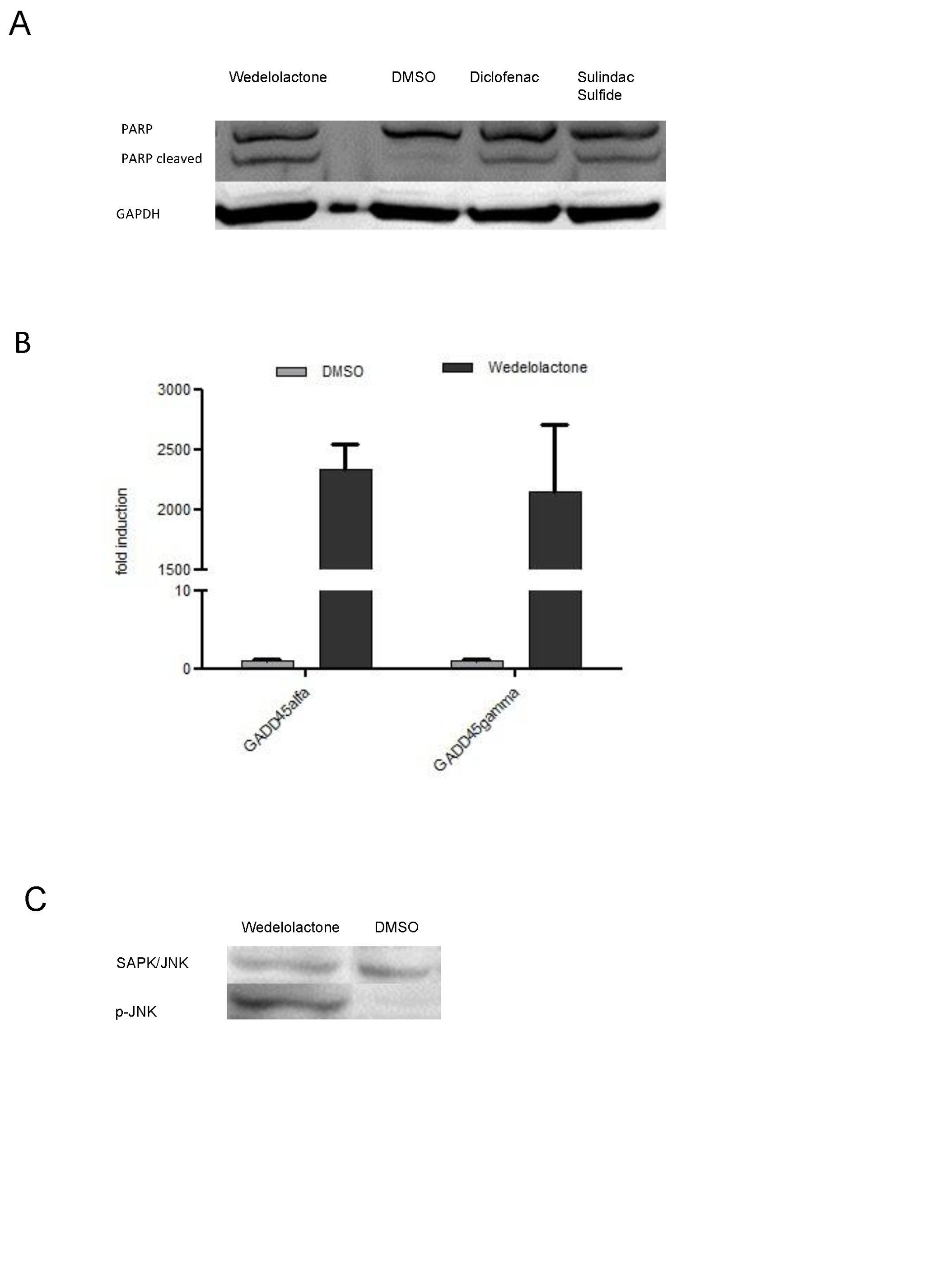

Supplement: Figure S4 — NSAIDs and NF-κB inhibitor activities in ovarian cancer cells. (A) NSAIDs and NF-κB inhibitors induce PARP activation. Western Blot analysis using anti PARP antibody (Santa Cruz) of cell lysates from CAOV-3 cells treated with 50 µM Sulindac Sulfide, 100 µM Diclofenac, 200 µM IKK inhibitor II Wedelolactone (7-Methoxy-5,11,12-trihydroxy-coumestan) or DMSO. (B) Real-time PCR analysis of CAOV-3 cells treated with 200 µM IKK inhibitor II Wedelolactone (7-Methoxy-5,11,12-trihydroxy-coumestan) or DMSO shows induced expression of GADD45 α and γ genes, (C) while western Blot analysis using anti-phospho JNK antibody of cell lysates from CAOV-3 cells treated with 200 µM IKK inhibitor II Wedelolactone (7-Methoxy-5,11,12-trihydroxy-coumestan) or DMSO shows activation of JNK. (TIF) [file pone.0024285.s004.tif]
