# Supplementary material for: Combinatorial Effect of Non-Steroidal Anti-inflammatory Drugs and NF-κB Inhibitors in Ovarian Cancer Therapy
Source: PLoS One. 2011 Sep 12;6(9):e24285. doi: 10.1371/journal.pone.0024285 (PMC3171406; doi:10.1371/journal.pone.0024285)
Supplement: Methods S1 — Description of Real Time methodology. (DOCX) [file pone.0024285.s005.docx]

### Supporting Methods S1

### Real-time PCR. Total RNA was harvested using QIAshredder (Qiagen, Valencia, CA) and RNeasy Mini kit (Qiagen). Real-time PCR was performed as described [[22](#_ENREF_22)]. cDNAs were generated from 2 μg of total RNA using Ready-to-Go You-Prime First-Strand Beads (Amersham Pharmacia Biotech, Inc., Piscataway, NJ). Amplifications of 0.1 μg cDNA were carried out using SYBR Green I–based real-time PCR on the MJ Research DNA Engine Opticon Continuous Fluorescence Detection System (MJ Research, Inc., Waltham, MA). All PCR mixtures contained PCR buffer [final concentration 10 mmol/L Tris-HCl (pH 9.0), 50 mmol/L KCl, 2 mmol/L MgCl_2_, and 0.1% TritonX-100], 250 μmol/L deoxynucleotide triphosphate (Roche, Indianapolis, IN), 0.5 μmol/L of each PCR primer, 0.5 x SYBR Green I (Molecular Probes, Eugene, OR), 5% DMSO, and 1 unit Taq DNA polymerase (Promega, Madison, WI) with 2 μL cDNA in a 25 μL final volume reaction mix. The samples were loaded into wells of low-profile, 96-well microplates. After an initial denaturation step of 60 seconds at 94°C, conditions for cycling were 40 cycles of 30 seconds at 94°C, 30 seconds at 52°C, and 1 minute at 72°C. Then, the fluorescence signal was measured right after incubation for 5 seconds at 79°C that follows the extension step, which eliminates possible primer dimer formation. At the end of the PCR cycles, a melting curve was generated to identify the specificity of the PCR product. For each run, serial dilutions of human glyceraldehyde-3-phosphate dehydrogenase (hGAPDH) plasmids were used as standards for quantitative measurement of the amount of amplified DNA. Also, for normalization of each sample, hGAPDH primers were used to measure the amount of hGAPDH cDNA. All samples were run in triplicates and the data were presented as gene-to-GAPDH ratio.

### Western-blot. Ovarian cancer cell lines were treated for 24 hours with different drugs and the whole-cell lysates of treated and control cells were prepared in lysis buffer. 100μg of the clarified lysate were denatured in 3X SDS sample buffer and proteins were resolved by SDS-10% PAGE and probed with antibodies against total SAPK/JNK (New England Biolabs), phospho JNK (Cell Signaling), *mda-7/*IL-24 (kindly provided by Paul Fisher, Virginia Commonwealth University, Richmond, VA ), PARP and GAPDH (Santa Cruz).
